# Gender disparity and post-traumatic stress disorder and elevated psychological distress in humanitarian migrants resettled in Australia: the moderating role of socioeconomic factors

**DOI:** 10.1017/S2045796024000489

**Published:** 2024-11-07

**Authors:** Demelash Woldeyohannes Handiso, Jacqueline A Boyle, Eldho Paul, Frances Shawyer, Joanne C Enticott

**Affiliations:** 1 Monash Centre for Health Research and Implementation, Faculty of Medicine, Nursing and Health Sciences, Monash University, Melbourne, Victoria, Australia; 2 Health Systems and Equity, Eastern Health Clinical School, Monash University, Melbourne, Victoria, Australia; 3 Southern Synergy, Department of Psychiatry, Monash University, Melbourne, Victoria, Australia

**Keywords:** common mental disorders, economic issues, gender differences, post-traumatic stress disorder, social factors

## Abstract

**Aims:**

Humanitarian migrants are at increased risk of post-traumatic stress disorder (PTSD) and elevated psychological distress. However, men and women often report varying degrees of stress and experience different challenges during migration. While studies have explored PTSD, psychological distress, gender, and resettlement stressors, they have not explored the interplay between these factors. This study aims to address that gap by investigating gender disparities in PTSD and psychological distress among humanitarian migrants in Australia, with a focus on the moderating role of socioeconomic factors.

**Methods:**

This study used data from five waves of the Building a New Life in Australia (BNLA) survey, a longitudinal study of 2,399 humanitarian migrants who arrived in Australia in 2013. PTSD and psychological distress were measured using the PTSD-8 and Kessler-6 (K6) scales, respectively. We conducted generalised linear mixed-effect logistic regression analyses stratified by gender.

**Results:**

Female humanitarian migrants exhibited a significantly higher prevalence of PTSD and psychological distress than males over five years of resettlement in Australia. Women facing financial hardship, unemployment, or residing in short-term housing reported greater levels of PTSD and distress compared to men.

**Conclusions:**

Women facing financial hardship, inadequate housing, and unemployment exhibit higher rates of PTSD and psychological distress, underscoring the significant impact of socioeconomic factors. Addressing these challenges at both individual and systemic levels is essential for promoting well-being and managing mental health among female humanitarian migrants.

## Introduction

A humanitarian migrant is an individual who relocates to another country due to being forcibly displaced or persecuted, often in search of refuge and protection (UNHCR, 2019). As per the United Nations High Commissioner for Refugees (UNHCR), the number of people forcibly displaced in 2022 rose by 19 million to reach 108.4 million, marking the largest increase between years ever recorded. Among these, 35.3 million were refugees, and nearly half were women (Freedman, 2016; UNHCR, 2022).

Throughout the phases of pre-migration, migration and post-migration, humanitarian migrants may encounter mental health risks (Wessels, 2014). These risk factors include exposure to traumatic events, stressors associated with the asylum process and challenges in establishing a new life (Wessels, 2014). During resettlement, humanitarian migrants confront various challenges such as language barriers, financial difficulties, social integration crises, discrimination and other issues (Hynie, 2018). The gender of humanitarian migrants moulds their migration experiences, influencing adaptation and mental health (Boyd and Grieco, 2003).

Psychological distress and mental disorders such as post-traumatic stress disorder (PTSD) in migrants have profound social implications (Perreira and Ornelas, 2013). Mental disorders lead to personal anguish and economic strain on society, contributing to healthcare expenses and decreased productivity. Untreated PTSD can hinder migrants’ integration, escalating social tensions and hindering overall societal advancement. Women may experience additional burdens due to gender-related societal roles, such as childcare responsibilities (Llácer *et al.*, 2007), weaker attachment to the labour market, more home-based roles and increased risk of sexual violence and abuse, contributing to the risk of development of conditions such as PTSD and elevated psychological distress (Vallejo-Martín *et al.*, 2021). Despite the high rates of PTSD and elevated psychological distress among female humanitarian migrants, only about one-quarter seek assistance, be it through professional or informal channels (Slewa-Younan *et al.*, 2015).

Factors including socioeconomic conditions contribute to the diminished mental well-being of humanitarian migrants in their host countries (Mwanri *et al.*, 2022). Moreover, social inequalities in poor mental health among female humanitarian migrants arise from the challenges experienced during migration and the uneven distribution of social determinants of health. This acknowledgment highlights the complexities involved in understanding these inequities, ultimately manifesting as disparities in the mental health outcomes of humanitarian migrants (Bowleg, 2012; Hollander, 2013; Viruell-Fuentes *et al.*, 2012). The overlap of being both a woman and a humanitarian migrant may consequently amplify disadvantages and the risk for developing mental disorders.

Despite the higher likelihood of PTSD and elevated psychological distress among female humanitarian migrants, there is limited research aiming to understand the primary drivers behind this trend (Due *et al.*, 2018; Sullivan *et al.*, 2020). This gap is particularly significant considering the increasing number of female humanitarian migrants resettling in Australia (Sullivan *et al.*, 2020), coupled with the rising population of such migrants with unmet mental health needs (O’Neil, 2019; Straiton *et al.*, 2014). This study assessed gender disparities in PTSD and elevated psychosocial distress among humanitarian migrants over time and aimed to identify the role of socioeconomic factors in the relationship between gender and PTSD, as well as elevated psychological distress. The findings from this research will enrich existing literature and provide insights to assist policymakers and service providers in developing more targeted interventions.

## Methods

### Data source, study participants and data collection procedure

We used data from ‘Building a New Life in Australia (BNLA): The Longitudinal Study of 2399 Humanitarian Migrants’, a unique data set from a nationally representative survey of humanitarian migrants from different cultural backgrounds who arrived in Australia and received permanent protection visas through Australia’s humanitarian programs between May and December 2013 (Australian Government: Department of Social Services, 2022). Data collectors acquired a list of all eligible participants from the Australian Government’s Department of Immigration and Border Protection and study participants provided consent to participate in the survey before data collection commenced. Data were collected longitudinally from 2013 to 2018 to increase understanding of the factors that determine how well humanitarian migrants build new lives in Australia in the first 5 years of resettlement (Australian Government: Department of Social Services, 2022).

The primary applicants were first asked to participate, and then other family members on the visa application were invited to join the study as secondary applicants (Australian Government: Department of Social Services, 2022). The survey tool was checked for internal consistency and translated into 19 languages to ensure that most participants could answer the survey in their primary language (Australian Government: Department of Social Services, 2022).

### Outcome variables

PTSD and elevated psychological distress were measured using the PTSD-8 (Hansen *et al.*, 2010) and Kessler-6 (K6) (Kessler *et al.*, 2002, 2003) scales, respectively. Adapted from the Harvard Trauma Questionnaire (HTQ), the PTSD-8 is a screening questionnaire that assesses the three PTSD clusters of intrusion, avoidance and hypervigilance according to the Diagnostic and Statistical Manual of Mental Disorders (DSM-IV) (Hansen *et al.*, 2010). It is as an effective screening tool for PTSD in vulnerable populations, including humanitarian migrants (Hansen *et al.*, 2010). The PTSD-8 scale utilizes a four-point rating system ranging from ‘not at all’ (1) to ‘most of the time’ (4), resulting in a total score between 8 and 32. The presence of PTSD is determined if at least one item in each symptom domain is rated 3 (sometimes) or 4 (most of the time). The K6 is a shortened version of the K10, scoring between 6 and 30. A score of 19 or higher indicates elevated psychological distress. The internal consistency of the tools was evaluated using Cronbach’s alpha, resulting in 0.96 and 0.93 for PTSD-8 and Kessler-6, respectively (Australian Bureau of Statistics, 2021).

### Covariates

Sociodemographic characteristics included age, marital status, level of education and gender. Socioeconomic factors included financial hardship, region of origin, housing arrangement and employment. Resettlement-related stressors include loneliness, trauma, experience of discrimination and chronic health conditions. See Supplementary file 1 for details.

### Data analysis

All analyses were conducted using Stata version 17 (StataCorp, Texas, USA). Population sample weights provided by the data custodians were applied to all analyses. The sociodemographic characteristics of the cohort were summarized using frequencies, percentages and 95% confidence intervals (CIs). The prevalence rate of both PTSD and elevated psychological distress were compared between males and females across the five waves. Potential variables were identified from the BNLA database by reviewing previous publications in the mental health area of humanitarian migrants (Cooper *et al.*, 2019; Wu *et al.*, 2021), and then the process of selecting variables for the final model involved the application of Least Absolute Shrinkage and Selection Operator (LASSO)adaptive methods. Cross-validations were employed to determine the alpha hyperparameters to ensure the optimal performance of the LASSO regression. Considering the longitudinal nature of the BNLA data where observations are non-independent and variances are unequal, the assumptions of the traditional logistic regression model are violated. Hence, a more suitable approach for this data set is a generalized linear mixed-effect logistic regression model. To explore whether socioeconomic factors moderate the association between gender and PTSD or elevated psychological distress, an interaction-effect analysis was conducted in the final model. Adjusted odds ratios and 95% CIs were presented in tables and line graphs, illustrating the moderating role of socioeconomic factors in the association between gender and PTSD and psychological distress. Results were deemed statistically significant at *p* < 0.05.

### Missing data management

The management of missing data involved a preliminary examination of its potential impact on outcome variables through logistic regression. In instances where a significant association between missing data and outcome variables was identified, multiple imputations analysis was employed. Multiple imputation is a statistical technique used to address missing data by generating multiple plausible values for each missing observation. We conducted 20 imputations to enhance robustness, mitigate bias and accommodate variability (Dong and Peng, 2013). We compared the data set without imputation to the data set after multiple imputations using Akaike Information Criteria (AIC) and the log-likelihood ratio. After imputation, we opted for the data set after imputations because it showed the lowest AIC and the highest log-likelihood ratio value, indicating the best-fit model.

### Ethical consideration

The BNLA data are publicly available to authorized researchers who have obtained permission from the Australian Government Department of Social Services. The Australian Institute of Family Studies ethics committee approved the data, which is registered with the National Health and Medical Research Council. The measures undertaken to ensure the truthfulness of responses to the government-administered survey included ensuring trust, confidentiality and anonymity, along with assurances against data misuse. Moreover, informed consent was obtained from all study participants involved in the BNLA study. Data were provided after signing a data usage and confidentiality deed. Demelash Handiso and Joanne Enticott obtained the permission to use the data set. The Monash University Human Research Ethics Committee advised that a separate ethics application wasn’t necessary since this secondary data analysis within the overall BNLA program had already received approval (number: 2017-8041-8528).

## Results

### Sociodemographic factors

Over half of humanitarian migrants were male, accounting for 54.0% of the total and the majority of participants were married. Most of the study participants were from the Middle East (58.5% of females and 53.5% of males) and Central Asia (23.1% of females and 25.5% of males). Approximately half of the females (47.3%) and males (48.8%) had completed 6–12 years of schooling, while 8.9% of females and 10.9% of males held a university degree (Table 1).

**Table 1.** Sociodemographic characteristics by gender

		Wave 1 (2399)		Wave 2 (1888)		Wave 3 (1767)		Wave 4 (1804)		Wave 5 (1780)	
Variables	Categories	Male *n* (%)	Female *n* (%)	Male *n* (%)	Female *n* (%)	Male *n* (%)	Female *n* (%)	Male *n* (%)	Female *n* (%)	Male *n* (%)	Female *n* (%)
Age category	15–25	283 (21.7)	260 (23.7)	260 (24.8)	224 (26.7)	192 (20.5)	186 (22.5)	180 (18.5)	169 (20.3)	154 (16.4)	149(17.7)
	26–35	582 (44.7)	480 (43.8)	525 (50.0)	409 (48.7)	475 (50.5)	408 (49.3)	488 (50.2)	411 (49.4)	456 (48.5)	421 (50.1)
	36–45	236 (18.1)	189 (17.3)	157 (14.9)	114 (13.6)	152 (16.1)	130 (15.7)	168 (17.3)	138 (16.6)	189 (20.1)	151 (17.9)
	46–55	122 (9.4)	100 (9.14)	68 (6.4)	63 (7.5)	78 (8.3)	69 (8.3)	86 (8.8)	69 (8.3)	82 (8.7)	75 (8.9)
	56–80	78 (6.0)	66 (6.03)	39 (3.7)	29 (3.5)	42 (4.4)	35 (4.2)	51 (5.2)	44 (5.3)	59 (6.3)	44 (5.2)
Marital status	Married	8596 (5.9)	603 (55.1)	672 (64.0)	453 (53.9)	634 (67.5)	446 (53.8)	645 (66.2)	464 (55.8)	653 (69.4)	494 (58.8)
	Separated	16 (1.2)	22 (2.0)	9 (0.8)	15 (1.8)	634 (67.5)	21 (2.5)	13 (1.3)	18 (2.1)	15 (1.6)	23 (2.7)
	Divorced	8 (0.6)	34 (3.11)	7 (0.6)	30 (3.5)	6 (0.6)	22 (2.6)	7 (0.7)	21 (2.5)	8 (0.8)	26 (3.1)
	Widowed	13 (1.0)	176 (16.1)	13 (1.2)	133 (15.8)	13 (1.3)	143 (17.2)	4 (0.4)	137 (16.4)	7 (0.7)	127 (15.1)
	Never married	405 (31.1)	259 (23.7)	348 (33.1)	208 (24.7)	267 (28.4)	196 (23.6)	304 (31.2)	191 (22.9)	257 (27.3)	170 (20.2)
Years of education	Never attended school	158 (12.1)	213 (19.5)	117 (11.2)	161 (19.1)	111 (11.8)	168 (20.2)	125 (12.8)	167 (20.1)	116 (12.3)	156 (18.5)
	<6 years school	272 (20.9)	208 (19.0)	216 (20.6)	150 (17.8)	205 (21.8)	165 (19.9)	202 (20.7)	156 (18.8)	199 (21.1)	168 (20.0)
	6–12 years of school	635 (48.8)	518 (47.3)	524 (49.9)	406 (48.3)	456 (48.5)	385 (46.5)	469 (48.2)	387 (46.5)	456 (48.5)	404 (48.1)
	Trade/Technical qualification	92 (7.1)	57 (5.16)	76 (7.2)	45 (5.3)	67 (7.1)	38 (4.5)	71 (7.3)	46 (5.5)	68 (7.2)	43 (5.1)
	University degree	142 (10.9)	97 (8.9)	116 (11.0)	77 (9.1)	100 (10.6)	72 (8.7)	106 (10.8)	75 (9.0)	101 (10.7)	69 (8.2)
Region of origin	Middle East	697 (53.5)	641 (58.5)	554 (52.8)	497 (59.2)	507 (53.9)	477 (57.6)	534 (54.8)	497 (59.8)	527 (56.0)	498 (59.2)
	Central Asia	332 (25.5)	253 (23.1)	258 (24.5)	180 (21.4)	241 (25.6)	199 (24.0)	256 (26.3)	201 (24.2)	224 (23.8)	191 (22.7)
	Southern Asia	143 (11.0)	79 (7.2)	127 (12.1)	63 (7.5)	105 (11.2)	61 (7.4)	100 (10.2)	65 (7.8)	100 (10.6)	60 (7.1)
	South-East Asia	57 (4.4)	63 (5.76)	51 (4.8)	56 (6.6)	40 (4.3)	51 (6.1)	34 (3.5)	31 (3.7)	40 (4.2)	50 (6.0)
	Africa	71 (5.44)	57 (5.23)	59 (5.6)	43 (5.1)	46 (4.9)	40 (4.8)	49 (5.0)	37 (4.4)	49 (5.2)	41 (4.9)

Sampling weights were applied to derive prevalence estimates

### The pattern of PTSD and psychological distress across the five waves

**Figure 1.**
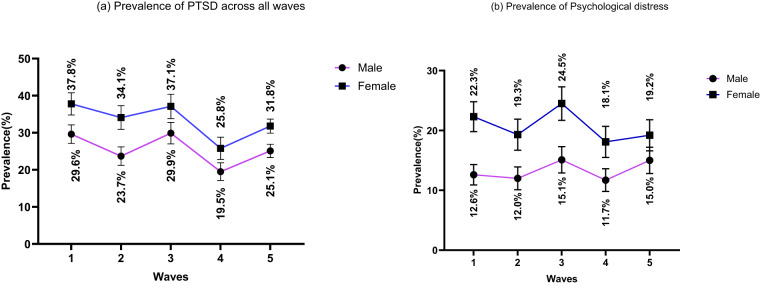
Prevalence (trend) of PTSD and elevated psychological distress in males and females across five waves, error bars denote a 95% confidence interval; sampling weights were applied to derive prevalence estimates. a) Prevalence of PTSD across all waves and b) Prevalence of Psychological distress across all waves.

The pattern of PTSD and psychological distress among humanitarian migrants was the same across the five waves. A high proportion of both females and males developed PTSD and psychosocial distress over time. However, the prevalence was consistently higher in females across all five waves of the study, although the magnitude of differences decreased over time. In wave 1, the prevalence of positive PTSD screening was 8.2% higher for females compared to males (37.7% vs 29.5%) and for elevated psychological distress the prevalence was 9.6% higher for females (22.3% vs 12.7%). By wave 5, female prevalence of PTSD was 31.9% compared to 25.2% for males, showing a difference of 6.7% while 19.2% of females and 15.0% of males showed elevated psychological distress, indicating a difference of 4.2% (Fig. 1aand b). The overall mean and standard deviation for the PTSD-8 and K6 were 15.5 ± 7.0 and 12.6 ± 5.9, respectively. In terms of PTSD, the specific values for avoidance were 4.0 ± 2.2, intrusion was 7.6 ± 3.6 and hypervigilance was 2.8 ± 1.9.

### Pre-arrival experiences of trauma in males and females

Compared to men, a higher proportion of women experienced extreme living conditions before arriving in Australia while men were more likely to experience violence, imprisonment/kidnapping, torture and serious injury. Similar proportions of men and women reported experiencing murder or disappearance of family members pre-arrival (Supplementary file 2).

### Factors associated with PTSD and elevated psychological distress in females and males

In univariate analysis, age, gender, geographical origin, financial hardship, chronic health conditions and experiences of discrimination and loneliness contributed to PTSD and elevated psychological distress among humanitarian migrants (Table 2). In final analysis, male humanitarian migrants over the age of 36 had an increased risk of PTSD compared to those in the 15–25 age group. Men from the Middle East were at a greater risk of developing PTSD than those from Africa. Furthermore, we found that each factor of financial hardship was significantly associated with an increase in both PTSD and elevated psychological distress for both genders. Self-reported chronic health conditions showed a negative association with psychological distress in both males and females, with a similar negative correlation observed for PTSD in males. Individuals who reported experiencing discrimination and loneliness were more likely to have psychological distress and PTSD compared to their counterparts (Table 3).

**Table 2.** Univariate analysis for the association between PTSD and psychological distress and determinant factors

		PTSD		PD	
		Male	Female	Male	Female
Variables		COR	COR	COR	COR
Age	15–25	1.0	1.0	1.0	1.0
	26–35	1.7 (1.4, 2.1)	1.6 (1.3, 1.9)	1.7 (1.3, 2.2)	1.6 (1.3, 1.9)
	36–45	3.2 (2.6, 4.0)	2.8 (2.3, 3.4)	2.8 (2.1, 3.7)	2.8 (2.3, 3.4)
	46–55	3.2 (2.4, 4.1)	3.2 (2.5, 4.0)	2.7 (1.9, 3.7)	3.2 (2.5, 4.0)
	56–80	3.3 (2.4, 4.3)	2.2 (1.6, 2.9)	2.2 (1.5, 3.2)	2.2 (1.6, 2.9)
Marital status	Married	1.0	1.0	1.0	1.0
	Separated	0.7 (0.4, 1.3)	0.8 (0.4, 1.2)	1.3 (0.7, 2.5)	2.2 (1.4, 3.4)
	Divorced	0.8 (0.3, 1.8)	0.3 (0.01, 0.7)	1.7 (0.7, 3.8)	1.4 (1.1, 2.1)
	Widowed	0.3 (0.1, 0.8)	0.4 (0.2, 0.5)	0.4 (0.1, 1.3)	1.4 (1.2, 1.7)
	Never married	0.5 (0.4, 0.6)	0.4 (0.5, 1.21)	0.6 (0.5, 0.8)	0.6 (0.5, 0.8)
Pre-arrival education	Never attended school	1.0	1.0	1.0	1.0
	<6 years of school	1.2 (0.9, 1.6)	1.1 (0.9, 1.3)	1.1 (0.8, 1.5)	1.1 (0.9, 1.3)
	6–12 years of school	1.4 (1.2, 1.8)	1.1 (0.9, 1.3)	1.2 (0.9, 1.6)	1.1 (0.9, 1.2)
	Trade/Technical qualification	2.3 (1.7, 3.2)	1.6 (1.2, 2.1)	1.8 (1.2, 2.6)	1.6 (1.2, 2.2)
	University degree	2.1 (1.6, 2.7)	1.2 (0.9, 1.5)	1.4 (1.02, 2.02)	1.2 (0.9, 1.5)
Origin of country	Middle East	1.9 (1.4, 2.6)	1.8 (1.4, 2.5)	2.3 (1.5, 3.5)	1.8 (1.3, 2.5)
	Central Asia	0.4 (0.3, 0.6)	1.1 (0.7, 1.4)	0.7 (0.4, 1.2)	1.0 (0.7, 1.4)
	Southern Asia	0.9 (0.6, 1.3)	0.6 (0.4, 0.9)	0.9 (0.5, 1.5)	0.6 (0.4, 0.9)
	South-East Asia	0.6 (0.3, 0.9)	0.4 (0.2, 0.6)	0.3 (0.2, 0.8)	0.4 (0.2, 0.6)
	Africa	1.0	1.0	1.0	1.0
Financial hardship	No	1.0	1.0	1.0	1.0
	1–2	1.9 (1.7, 2.2)	1.7 (1.5, 2.0)	2.5 (2.1, 3.0)	1.7 (1.5, 2.02)
	3–4	2.5 (2.1, 3.1)	2.8 (2.3, 3.4)	4.7 (3.7, 5.9)	2.8 (2.3, 3.4)
	5–6	2.8 (1.9, 4.1)	3.4 (2.3, 4.9)	5.2 (3.5, 7.8)	3.4 (2.3, 4.9)
Current employment	Yes	1		1.3 (1.0, 1.6)	1.4 (1.1, 1.7)
	No	1.6 (1.3, 1.9)	1.35 (1.0, 1.7)	1.0	1.0
Chronic health condition	Yes	2.4 (2.1, 2.8)	1.8 (1.6, 2.1)	4.5 (3.7, 5.5)	3.3 (2.7, 3.9)
	No	1.0	1.0	1.0	1.0
Loneliness	Yes	1.49 (1.3, 1.7)	1.8 (1.5, 2.1)	2.03 (1.7, 2.4)	1.8 (1.5, 2.2)
	No	1.0	1.0	1.0	1.0
Community support	Yes	1.0	1.0	1.0	1.0
	Some time	1.2 (1.04, 1.4)	1.3 (1.1, 1.5)	0.8 (0.7, 1.0)	1.3 (1.1, 1.5)
	No	1.1 (0.9, 1.2)	1.2 (1.1, 1.4)	0.7 (0.6, 0.9)	1.2 (1.1, 1.4)
Trauma	No	1.0	1.0	1.0	1.0
	Yes	2.2 (1.7, 2.9)	3.4 (2.6, 4.6)	2.1 (1.4, 3.1)	3.4 (2.6, 4.6)
Experience of discrimination	Yes	1.5 (1.2, 1.9)	1.0	1.9 (1.4,2.4)	1.5 (1.2, 1.9)
	No	1.0	0.6 (0.5, 0.8)	1.0	1.0

COR: crude odds ratio; sampling weights were applied to derive odds ratios.

**Table 3.** Factors associated with psychological distress and PTSD stratified by gender of humanitarian migrants

		Elevated psychological distress		Post-traumatic stress disorder	
Variables		Male (AOR)	Female (AOR)	Male (AOR)	Female (AOR)
Age	15–25		1	1	
	26–35	1.1 (0.6, 1.6)	1.1 (0.7, 1.6)	1.3 (0.9, 1.8)	0.4 (0.3, 0.6)
	36–45	1.1 (0.6, 1.8)	1.2 (0.8, 1.9)	2.1 (1.4, 3.2)*	0.5 (0.4, 0.7)
	46–55	0.9 (0.5, 1.8)	1.1 (0.6, 1.8)	2.3 (1.5, 3.5)*	0.8 (0.6, 1.3)
	56–80	0.8 (0.4, 1.7)	0.9 (0.4, 1.8)	2.2 (1.3, 3.6)*	0.6 (0.4, 1.1)
Marital status	Married	1	1	1	
	Separated	2.2 (0.7, 6.5)	1.5 (0.7, 3.0)	0.9 (0.4, 2.1)	1.2 (0.6, 2.2)
	Divorced	1.4 (0.4, 5.2)	1.3 (0.6, 2.6)	1.1 (0.3, 3.8)	1.4 (0.8, 2.4)
	Widowed	1.4 (0.6, 3.6)	0.3 (0.1, 0.9)	0.6 (0.2, 2.2)	1.1 (0.8, 1.5)
	Never married	1.2 (0.8, 1.7)	0.2 (0.03, 0.7)	0.8 (0.6, 1.0)	0.6 (0.5, 1.9)
Pre-arrival education	Never attended school	1	1	1	
	<6 years of school	0.8 (0.4, 1.4)	1.1 (0.7, 1.7)	0.8 (0.6, 1.3)	1.2 (0.8, 1.7)
	6–12 years of school	0.9 (0.5, 1.6)	1.1 (0.7, 1.8)	0.9 (0.6, 1.4)	1.1 (0.7, 1.6)
	Trade/Technical qualification	1.0 (0.5, 2.1)	1.2 (0.6, 2.2)	0.9 (0.5, 1.7)	1.8 (0.9, 3.4)
	University degree	0.8 (0.4, 1.6)	1.4 (0.8, 2.6)	1.0 (0.6, 1.6)	1.3 (0.7, 2.4)
Region of origin	Middle East	2.9 (1.2, 6.9)*	1.5 (0.7, 3.0)	2.9 (1.8, 4.8)*	2.2 (1.2, 3.9)*
	Central Asia	1.4 (0.5, 3.7)	1.3 (0.6, 2.5)	0.7 (0.4, 1.3)	1.4 (0.7, 2.6)
	Southern Asia	1.2 (0.5, 3.0)	0.3 (0.1, 1.9)	1.9 (1.1, 3.4)*	1.2 (0.5, 2.5)
	South-East Asia	0.8 (0.2, 2.7)	0.2 (0.1, 1.7)	0.8 (0.4, 2.0)	0.4 (0.1, 1.8)
	Africa	1	1	1	
Financial hardship	No	1	1	1	1
	1–2	1.9 (1.5, 2.6)***	1.5 (1.1, 1.9)***	1.8 (1.4, 2.2)***	1.6 (1.3, 2.1)***
	3–4	3.7 (2.5, 5.3)***	2.8 (1.9, 4.2)***	1.9 (1.4, 2.8)***	3.3 (2.3, 4.5)***
	5–6	4.4 (2.3, 8.5)***	2.4 (1.2, 4.6)***	3.3 (1.9, 5.7)***	2.5 (1.4, 4.6)***
Housing	Own	1			
	Temporary	4.8 (1.6, 14.2)*	1.5 (0.5, 4.5)	8.2 (3.1, 22.3)*	2.5 (0.9, 6.4)
	Short-term lease/contract	3.1 (1.1, 8.8)*	0.9 (0.3, 2.4)	8.9 (3.4, 23.7)*	2.1 (0.9, 5.1)
	Long-term lease/contract	2.6 (0.9, 7.1)	0.8 (0.3, 1.9)	6.9 (2.7, 18.2)*	1.3 (0.6, 2.9)
Current employment	Yes	1	1	1	1
	No	0.7 (0.5, 1.2)	0.8 (0.5, 1.4)	1.1 (0.8, 1.5)	1.1 (0.7, 1.8)
Chronic health condition	Yes	1.5 (1.1, 1.9)***	1.2 (0.9, 1.5)	1.8 (1.5, 2.2)***	1.6 (1.3, 1.9)***
	No	1	1	1	1
Loneliness	Yes	1.7 (1.3, 2.3)***	2.0 (1.6, 2.7)***	1.9 (1.5, 2.4)***	1.5 (1.2, 2.0)***
	No	1	1	1	1
Trauma	Yes	1.8 (1.1, 3.1)***	1.5 (0.9, 2.7)	1.9 (1.5, 2.4)***	2.6 (1.5, 4.2)***
	No	1	1	1	1
Experience of discrimination	Yes	1.9 (1.2, 2.9)***	1.7 (1.1, 2.7)***	1.7 (1.2, 2.3)***	1.7 (1.1, 2.8)***
	No	1	1	1	1
Received support	Yes	1	1	1	1
	Sometimes	1.2 (0.8, 1.6)	1.9 (0.6, 1.3)	1.5 (1.2, 2.0)*	1.6 (1.1, 2.3)*
	No	1.1 (0.8, 1.5)	0.8 (0.5, 1.2)	1.3 (0.9, 1.6)	1.4 (0.9, 1.9)

AOR: adjusted odds ratio; sampling weights were applied to derive AOR estimates.

* *p* < 0.05, ***p < 0.001.

**Figure 2.**
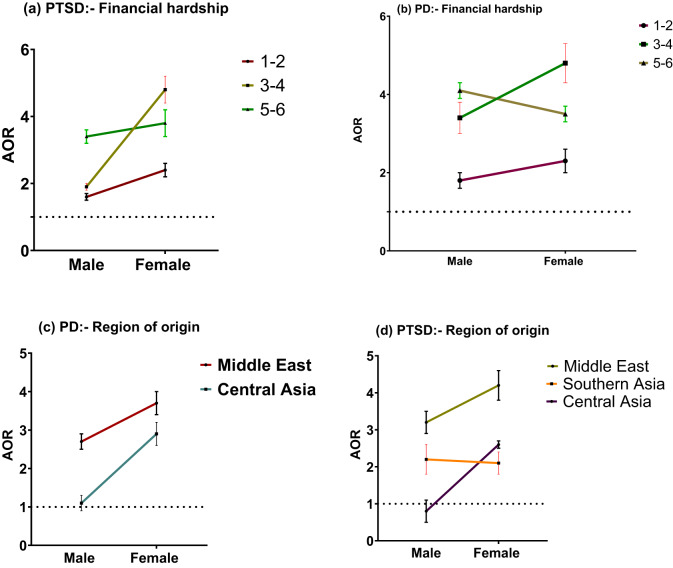
The moderation role of financial hardship and region of origin on the association between gender and PTSD and psychological distress (PD); error bars denote 95% confidence interval. (a) Moderating role of financial hardship in association between PTSD and gender; (b) Moderating role of financial hardship for association between Psychological distress and gender; (c) Moderating role of region of origin in association between Psychological distress and gender (d) Moderating role of region of origin in association between PTSD and gender. For example: Odds of developing PTSD for women with financial hardship levels of 5–6, 3–4 and 1–2 higher than men with the same financial hardship categories.

**Figure 3.**
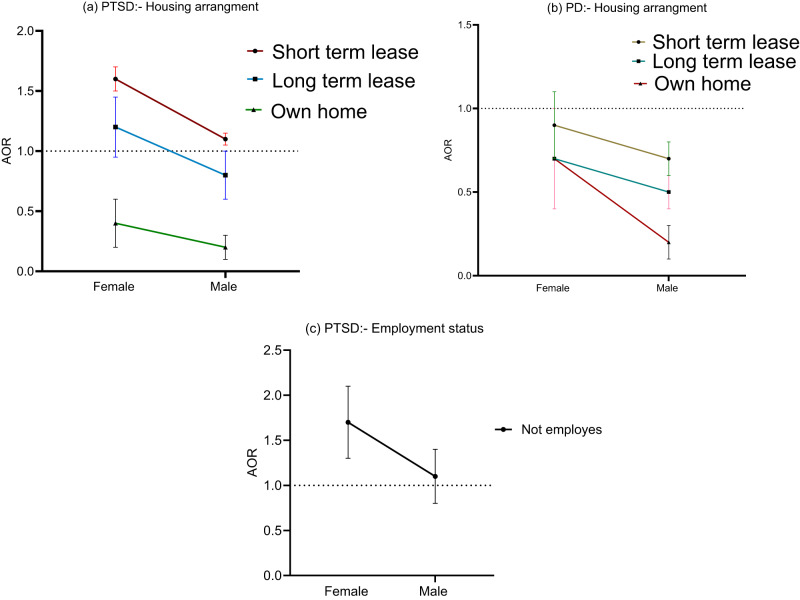
The moderation role of housing arrangement, employment status and support from religious groups on the association between gender and PTSD and psychological distress (PD); error bars denote a 95% confidence interval. (a) Moderating role of housing arrangement in association between PTSD and gender; (b) Moderating role of housing arrangement in association between Psychological distress and gender; (c) Moderating role of employment status in association between PTSD and gender.

In relation to socioeconomic moderators for gender and PTSD, humanitarian migrants experiencing financial hardship were more likely to experience PTSD, with women having a greater likelihood than men (Table 4). Women with financial hardship levels of 5–6, 3–4 and 1–2 had higher odds of developing PTSD compared to men with the same financial hardship levels, with specific odds ratios of (OR: 3.8; 95% CI: 3.4–4.2) and (OR: 3.4; 95% CI: 3.1–3.6), (OR: 4.8; 95% CI: 4.4–5.2) and (OR: 1.9; 95% CI: 1.8–2.0), and (OR: 2.4; 95% CI: 2.2–2.6) and (OR: 1.6; 95% CI: 1.5–1.7), respectively (Fig. 2a). Housing arrangement was another moderator for the association between PTSD and gender. Specifically, women (OR: 1.6; 95% CI: 1.5–1.7) in short-term lease housing reported higher odds of PTSD compared to men (OR: 1.1; 95% CI: 1.05–1.15) in similar housing arrangements (Fig. 3a). Although unemployment rates showed a slight decline from the first to the fifth wave, shifting from 38.6% (95% CI: 35.5–41.7) to 32.4% (95% CI: 29.15–35.8), employment status magnified gender disparities in PTSD. Unemployed women had a higher risk of PTSD compared to unemployed men, showing odds of (OR: 1.7; 95% CI: 1.3–2.1) (Fig. 3c). Females from the Middle East and Central Asia displayed higher odds of PTSD compared to male humanitarian migrants from the same region ((OR: 4.2; 95% CI: 3.8–4.6) vs (OR: 3.2; 95% CI: 2.9–3.5); (OR: 2.6; 95% CI: 2.5–2.7) vs (OR: 0.8; 95% CI: 0.5–1.1), respectively), while men from South Asia exhibited higher odds of PTSD compared to women from South Asian countries (Fig. 2d).

**Table 4.** Role of socioeconomic factors for association between gender and PTSD and elevated psychological distress

		IE-PTSD			IE-PD		
		Male	Female		Male	Female	
Variables		AOR	AOR	IE *p*-value for PTSD	AOR	AOR	IE *p*-value for PD
Age	15–25	1.0	1.3 (0.9, 1.9)	0.0132			0.088
	26–35	1.1 (0.8, 1.5)	1.5 (1.1, 2.1)*				
	36–45	1.4 (0.9, 2.1)	2.1 (1.4, 3.2)*				
	46–55	1.3 (0.9, 2.1)	1.6 (1.1, 2.6)*				
	56–80	1.3 (0.8, 2.1)	1.3 (0.7, 2.3)				
Marital status	Married	1.0		0.044			0.079
	Separated	0.8 (0.4, 1.8)	1.1 (0.5, 2.3)				
	Divorced	1.1 (0.3, 4.2)	1.4 (0.8, 2.5)				
	Widowed	0.8 (0.2, 2.7)	1.3 (0.9, 1.7)				
	Never married	0.8 (0.6, 1.1)	1.1 (0.8, 1.5)				
Pre-arrival education	Never attended school	1.0	1.0	0.32			0.202
	<6 years of school	0.9 (0.6, 1.4)	1.6 (0.1, 2.5)				
	6–12 years of school	1.2 (0.8, 1.8)	1.4 (0.9, 2.1)				
	Trade/Technical qualification	1.3 (0.7, 2.2)	2.5 (0.4, 4.6)				
	University degree	1.4(0.8, 2.2)	2.1(0.2, 3.6)				
Region of origin	Middle East	3.2 (2.9, 3.5)***	4.2 (3.8, 4.6)***	<0.001	2.7 (2.5, 2.9)***	3.7 (3.4, 4.0)***	<0.001
	Central Asia	0.8 (0.5, 1.1)	2.6 (2.5, 2.7)***		1.1 (0.6, 1.4)	2.9 (2.6, 3.2)***	
	Southern Asia	2.2 (1.8, 2.6)***	2.1 (1.8, 2.5)***		1.3 (0.5, 3.1)	0.8 (0.3, 2.3)	
	South-East Asia	0.8 (0.3, 2.1)	0.7 (0.3, 1.7)		0.7 (0.2, 2.3)	0.4 (0.1, 2.1)	
	Africa	1.0	1.0			1.0	
Financial hardship	No	1.0	1.0	<0.001			<0.001
	1–2	1.6 (1.5, 1.7)***	2.4 (2.2, 2.6)***		1.7 (1.5, 1.9)***	2.3 (2.0, 2.6)***	
	3–4	1.9 (1.8, 2.0)***	4.8 (4.4, 5.2)***		3.4 (3.0, 3.8)***	4.8 (4.3, 5.3)***	
	5–6	3.4 (3.1, 3.6)***	3.8 (3.4, 4.2)***		4.1 (3.9, 4.3)***	3.5 (3.3, 3.7)***	
Current employment	Yes	1.0		<0.001			0.073
	No	1.1 (0.8, 1.4)	1.7 (1.3, 2.1)***				
Chronic health condition	Yes	1.3 (1.1, 1.6)***	1.7 (1.3, 2.2)***	<0.001	1.4 (1.1, 1.8)***	1.8 (1.4, 2.4)***	0.0007
	No	1.0	1.0			1.0	
Loneliness	Yes	1.6 (1.3, 2.1)***	1.9 (1.5, 2.6)***	<0.001	1.7 (1.3, 2.2)***	2.8 (2.1, 3.8)***	0.007
	No	1.0	1.0		1.0	1.0	
Support from community group	Yes	1.0	1.0	0.0004			0.058
	Some time	1.7 (1.6, 1.8)*	1.9 (1.8, 2.0)*				
	No	1.5 (1.4, 1.6)*	1.5 (1.4, 1.6)*				
Housing	Own	0.2 (0.1, 0.3)***	0.4 (0.2, 0.6)***		0.7(0.6, 0.8)	0.2(0.1, 0.3)	
	Short-term lease/contract	1.1 (1.0, 1.2)	1.6 (1.5, 1.7)***		0.7 (0.6, 0.8)***	0.9 (0.7, 1.1)	
	Long-term lease/contract	0.8 (0.6, 1.0)	1.2 (0.9, 1.5)		0.5 (0.4, 0.6)***	0.7 (0.4, 1.0)	
Trauma	No	1.0	1.0	0.0063	1.0	1.0	0.001
	Yes	1.8 (1.2, 2.7)*	2.5 (1.6, 3.7)*		1.6 (0.9, 2.7)	2.3 (1.3, 3.7)	
Experience of discrimination	Yes	1.6 (1.2, 2.3)*	2.2 (1.4, 3.4)*	0.0087	1.9 (1.2, 2.9)***	2.2 (1.4, 3.6)***	0.0001
	No	1.0	1.0		1.0	1.0	

AOR: adjusted odds ratio; IE: interaction effect; PD: elevated psychological distress; PTSD: post-traumatic stress disorder; sampling weights were applied to derive AOR estimates.

* *p* < 0.05, ***p < 0.001.

Women facing financial hardship levels of 1–2 and 3–4 showed higher odds of elevated psychological distress compared to men ((OR: 4.8; 95% CI: 4.3–5.3) and (OR: 3.4; 95% CI: 3.0–3.8), (OR: 2.3; 95% CI: 2.0–2.6) and (OR: 1.7; 95% CI: 1.5–1.9), respectively). However, men experiencing financial hardship at level 5–6 exhibited higher odds of elevated psychological distress compared to females ((OR: 4.1; 95% CI: 3.9–4.3) and (OR: 3.5; 95% CI: 3.3–3.7), respectively) (Fig. 2b). Housing arrangements significantly moderated elevated psychological distress for males. Specifically, among men, those residing in long-term lease housing arrangements or their own houses had a reduced risk of elevated psychological distress, showing odds of (OR: 0.5; 95% CI: 0.4–0.7) and (OR: 0.2; 95% CI: 0.1–0.3), respectively (Fig. 3b). Women from the Middle East faced a higher risk of elevated psychological distress compared to men from the same region ((OR: 3.7; 95% CI: 3.4–4.0) and (OR: 2.7; 95% CI: 2.5–2.9), respectively). Similarly, women from Central Asia faced a higher risk of elevated psychological distress relative to men from the same region ((OR: 2.9; 95% CI: 2.6- 3.2) and (OR: 1.1; 95% CI: 0.6–1.4), respectively) (Fig. 2c).

## Discussion

### Overall findings

The escalating influx of humanitarian migrants to high income countries highlights the urgent need for a nuanced understanding of the social determinants impacting their mental health (Bambra *et al.*, 2005; Compton and Shim, 2015; Landau and Achiume, 2017). In this study, we investigated gender disparities in PTSD and elevated psychological distress among humanitarian migrants from 2013 to 2018 longitudinally including the moderating role of socioeconomic factors. We found a higher prevalence of PTSD and elevated psychological distress among females compared to males. In the first wave, PTSD prevalence was 37.7% for females and 29.5% for males, with elevated psychological distress rates of 22.3% and 12.7%, respectively. By the last wave, PTSD prevalence decreased to 31.9% for females and 25.2% for males, while psychological distress rates were 19.2% for females and 15.0% for males.

Socioeconomic factors such as financial hardship, housing arrangements and region of origin moderated the association between gender and mental health. Women facing socioeconomically disadvantaged circumstances, such as moderate financial hardship and unemployment, exhibited higher rates of both PTSD and elevated psychological distress compared to men with similar disadvantages. However, men in short-term housing arrangements or experiencing high levels of financial hardship displayed increased odds of experiencing elevated psychological distress when compared to females in the same circumstances.

The higher prevalence of PTSD and elevated psychological distress in female compared to male humanitarian migrants aligns with the pattern observed in the general population (Enticott *et al.*, 2022; Olff, 2017; Silove *et al.*, 2017), although here absolute rates are lower for both sexes. Research indicates that gender disparities in mental health are rooted in social factors, particularly variations in gender roles and expectations (Van Droogenbroeck *et al.*, 2018). These gendered patterns in social roles and positions arise due to unequal access to power and prestige, leading to disparities in opportunities, a disparity likely exacerbated within humanitarian migrant groups, especially in light of the impact of trauma (Hollander *et al.*, 2011; Jarallah and Baxter, 2019). Addressing these social factors is essential for promoting better mental health outcomes and fostering greater gender equality within migrant communities (Mirowsky and Ross, 2003; Olff, 2017; Tolin and Foa, 2008).

Our research underscores financial hardship as a risk factor for PTSD and elevated psychological distress among humanitarian migrants, particularly impacting women. This finding resonates with previous studies conducted in Canada and the United States (Bulut and Brewster, 2021; Tang *et al.*, 2007). Financial hardship can introduce various stressors among humanitarian migrants, including challenges in meeting basic needs, housing insecurity and social isolation, all contributing to the development of PTSD and elevated psychological distress (Torlinska *et al.*, 2020). Interventions that effectively target financial stressors can decrease the likelihood of mental health issues and improve overall well-being (Sullivan *et al.*, 2020).

A stable housing arrangement is a critical factor in alleviating mental health issues among males. Bulut and Brewster’s study observed that higher income and homeownership are linked to lower levels of mental health issues among migrants (Bulut and Brewster, 2021). In contrast, temporary housing contributes to stress, particularly for humanitarian migrants resettled in host countries. These findings indicate that short-term lease/temporary housing arrangements may be a contributing factor to the persistence of elevated psychological distress. This suggests that implementing policies that prioritize stable and secure housing options for migrants such as longer-term leases or other affordable housing options could alleviate psychological distress.

Both men and women from the Middle East had higher rates of PTSD than those from other regions in contrast to the findings from a systematic review that indicated that migrants from Africa experienced a higher rate of PTSD (Blackmore *et al.*, 2020). This disparity suggests the need for further investigation to clarify the effect of the migrants’ region of origin on their mental health in Australia. We also found that the region of origin serves as a moderator in the relationship between gender and mental disorders in humanitarian migrants. Women from the Middle East and Central Asia exhibited a higher likelihood of experiencing elevated psychological distress and PTSD compared to men from the same regions. These findings align with previous research highlighting the influence of the migrants’ region of origin in shaping the mental health of women who have resettled in Australia (Cooper *et al.*, 2019; Fozdar and Hartley, 2013). Our findings also align with a scoping review and a cross-sectional study conducted on migrant women from Middle Eastern countries in the United States (Almutairi *et al.*, 2022; Bulut and Brewster, 2021) as well as research on humanitarian migrants resettled in high-income countries showing that women from Middle Eastern countries exhibit a higher magnitude of PTSD compared to migrants from other regions. The observed associations may be linked to the higher likelihood of trauma and discrimination experienced by women from these regions, historically marked by prolonged conflict and war (Tolin and Foa, 2008). Understanding the impact of the country of origin on the development of elevated psychological distress holds substantial implications for preventing and treating mental health issues among humanitarian migrants.

Understanding migrants’ experiences throughout the migration process is crucial for crafting effective interventions. This involves recognizing and addressing challenges such as cultural stereotyping, discrimination, social integration hurdles, trauma impacts and issues specific to the region of origin.

### The limitations in previous research addressed by our study

While previous research has often focused on specific countries of origin, our study adopted a more inclusive approach, focusing on migrants from diverse regions worldwide. Unlike previous BNLA studies, which often had limited scope, examining only specific combinations or a reduced number of waves, our investigation encompassed all five waves within the database (Chen *et al.*, 2017, 2019; Cooper *et al.*, 2019; Nguyen *et al.*, 2023; Stuart and Nowosad, 2020; Wu *et al.*, 2021). While prior studies largely overlooked the critical role of socioeconomic factors in moderating the association between gender and mental disorders (Blackmore *et al.*, 2020), our research filled this gap. A major strength of our paper compared to previous BNLA publications was the selection process of covariates involving LASSO regression to identify the key associated factors from the large number of available variables.

### Policy implications

Our findings demonstrate that socioeconomic factors play a pivotal role in shaping mental health, closely aligning with the ongoing concerns of policymakers in Australia. This resonance underscores the urgency for implementing targeted interventions that address the needs of high-risk populations within the Australian policy framework (Australian Department of Health, 2017). In crafting effective policies to address mental illness, it’s imperative for policymakers and politicians to recognize and address the intricate interplay between social factors and mental well-being. Our study highlights the important role of socioeconomic factors in shaping the relationship between gender and mental health among humanitarian migrants establishing new lives in Australia. To improve the mental well-being of immigrant women, our findings suggest a re-evaluation of intervention strategies that target socioeconomic factors and address gender-specific issues impacting women’s mental health. A crucial focus should be on empowering immigrant women by providing enhanced employment opportunities, strengthening connections to the resettled country settings and taking steps to alleviate financial hardship, such as through economic support and social welfare programs. Prioritizing structural changes in the development of inclusive and gender-sensitive mental health initiatives can help mitigate the influence of these factors on the mental well-being of migrant women, promote equitable access to resources and foster a positive, supportive environment conducive to their overall mental health.

### Strengths and limitations

Our study builds upon previous research, utilizing high-quality data from humanitarian migrants resettled in Australia (Australian Government: Department of Social Services, 2022). A key strength lies in its comprehensive gender-stratified analysis of 5 years of follow-up data to assess mental disorders among both sexes of humanitarian migrants. By delving into the moderating role of socioeconomic factors in the relationship between gender and mental illness, we offer insights for policymakers and program planners seeking to design interventions to address mental disorders. However, the study also had certain limitations. Data collection methods varied across waves; the first, third and fifth waves used computer-assisted self-interviews, while the second and fourth waves relied on telephone interviews. This inconsistency poses a potential for bias due to the differences in self-reporting methods. Furthermore, this study is constrained by the temporal scope of the employment status measurement, which is limited to the last 7 days. This narrow timeframe may not adequately capture the broader employment patterns and their potential impact on the mental health of humanitarian migrants. Moreover, the study utilized the K6 instead of the K10, potentially leading to a loss of information and a more limited screening of mental illness. Although multiple imputations have been implemented to handle missing data, we did not conduct a sensitivity analysis, which we acknowledge as a limitation of our study. This also study did not consider non-binary gender identities.

### Conclusion

Comprehensively understanding PTSD, elevated psychological distress, gender disparities and the diverse influencing factors – especially socioeconomic aspects – among migrant women is crucial for effectively addressing their mental health needs following resettlement. Our research findings emphasize gender differences in PTSD and elevated psychological distress rates over the 5 years after resettlement in Australia, with socioeconomic factors playing a moderating role. Prioritizing efforts aimed at alleviating the socioeconomic stressors would provide crucial support for the mental well-being of female immigrants as they adapt to their new homes and help ensure a more positive and successful transition to their new lives.

## Supporting information

Handiso et al. supplementary material 1 Handiso et al. supplementary material

Handiso et al. supplementary material 2 Handiso et al. supplementary material

## Data Availability

The BNLA data are available for authorized people at the National Centre for Longitudinal Data website.

## Supplementary material

The supplementary material for this article can be found at https://doi.org/10.1017/S2045796024000489.
