# Supplementary material for: Gender disparity and post-traumatic stress disorder and elevated psychological distress in humanitarian migrants resettled in Australia: the moderating role of socioeconomic factors
Source: Epidemiol Psychiatr Sci. 2024 Nov 7;33:e60. doi: 10.1017/S2045796024000489 (PMC11561526; doi:10.1017/S2045796024000489)
Supplement: Handiso et al. supplementary material 1 — Handiso et al. supplementary material [file S2045796024000489sup001.docx]

**Supplementary file 1:** Defentions for the covariates

| **Covariates** | **Questions** |
| --- | --- |
| Financial hardship | Six questions queried issues such as delayed bills or rent payments and instances of going without meals due to financial constraints in the past year. The responses were grouped into four categories based on the number of financial stressors endorsed: 0, 1-2, 3-4, and 5-6. |
| Region of origin | The individual's country of birth is a proxy for nationality and is categorised according to the classification of the Australian Bureau of Statistics (Australian Bureau of Statistics, 2016) |
| Housing arrangement | Housing arrangement was measured using four choices: temporary (e.g., temporary/no contract), short-term lease/contract (i.e., less than six months), long-term lease/contract (i.e., more than six months), and own house. |
| Employment status | Those who had worked and received payment during this period were classified as 'employed,' while those who hadn't engaged in paid work were categorised as 'unemployed.' |
| Loneliness | Loneliness was evaluated by inquiring, 'Have any of the following been a source of stress in your life in the last 12 months?' The response options included 'loneliness (e.g., homesickness, lack of social life and/or friends)' and 'getting used to life in Australia.' |
| Experience of discrimination | The experience of discrimination was measured through the item: 'In the last 12 months, do you think you have been discriminated against, stopped doing something, or been hassled or made to feel inferior because of your ethnicity, religion, or skin colour?' |
| Chronic health conditions | Chronic health conditions were determined by asking, 'Do you have a disability, injury, or health condition that has lasted or is likely to last 12 months or more?' Responses for these factors were binary: 'yes' or 'no'. |
