# Supplementary material for: Gender disparity and post-traumatic stress disorder and elevated psychological distress in humanitarian migrants resettled in Australia: the moderating role of socioeconomic factors
Source: Epidemiol Psychiatr Sci. 2024 Nov 7;33:e60. doi: 10.1017/S2045796024000489 (PMC11561526; doi:10.1017/S2045796024000489)
Supplement: Handiso et al. supplementary material 2 — Handiso et al. supplementary material [file S2045796024000489sup002.docx]

**Supplementary file 2:** Mental health-related factors across the five waves

| **Variables** |  | **Wave 1** | | **Wave 2** | | **Wave 3** | | **Wave 4** | | **Wave 5** | |
| --- | --- | --- | --- | --- | --- | --- | --- | --- | --- | --- | --- |
|  |  | **Male n (%)** | **Female n (%)** | **Male n (%)** | **Female n (%)** | **Male n (%)** | **Female n (%)** | **Male n (%)** | **Female n (%)** | **Male n (%)** | **Female n (%)** |
| Experience of discrimination | Yes | 56 (4.5) | 52 (5.1) | 78 (7.7) | 69 (8.8) | 74 (8.0) | 62 (7.6) | 91 (9.4) | 81 (9.8) | 78 (8.4) | 59 (7.2) |
| Chronic health condition | Yes | 303 (24.3) | 270 (26.0) | 319 (30.4) | 306 (36.5) | 279 (29.7) | 266 (32.1) | 296 (30.4) | 319 (38.4) | 220 (23.4) | 315 (37.5) |
| Loneliness | Yes | 218 (17.8) | 179 (17.8) | 401 (46.9) | 166 (46.6) | 172 (19.2) | 121 (15.5) | 192 (25.2) | 114 (31.6) | 140 (15.5) | 115 (14.3) |
| Employment | Yes | 110 (8.8) | 84 (8.0) | 159 (15.2) | 23 (2.74) | 152 (16.2) | 44 (5.3) | 201 (20.7) | 48 (5.7) | 207 (22.0) | 63 (7.5) |
| Community support | Yes | 590 (49.1) | 442 (44.6) | 464 (45.8) | 365 (45.2) | 478 (51.5) | 376 (47.0) | 482 (50.5) | 415 (51.3) | 502 (54.3) | 408 (49.5) |
|  | Sometimes | 223 (18.5) | 199 (20.1) | 226 (22.3) | 173 (21.4) | 210 (22.7) | 175 (21.9) | 215 (22.5) | 169 (20.9) | 211 (22.8) | 191 (23.1) |
|  | No | 387 (32.3) | 350 (35.3) | 322 (31.8) | 269 (33.3) | 239 (25.7) | 249 (31.1) | 256 (26.8) | 225 (27.8) | 212 (22.9) | 225 (27.3) |
| Financial hardship | No | 742 (59.5) | 574 (55.1) | 459 (43.8) | 316 (37.7) | 533 (56.8) | 429 (51.8) | 597 (61.4) | 427 (51.4) | 662 (70.4) | 518 (61.6) |
|  | 1-2 | 365 (29.3) | 338 (32.5) | 383 (36.5) | 339 (40.4) | 270 (28.8) | 254 (30.6) | 261 (26.8) | 256 (30.8) | 200 (21.2) | 228 (27.1) |
|  | 3-4 | 115 (9.2) | 109 (10.4) | 180 (17.1) | 156 (18.6) | 103 (10.9) | 119 (14.4) | 87 (8.9) | 117 (14.1) | 60 (6.3) | 74 (8.8) |
|  | 5-6 | 25 (2.0) | 19 (1.8) | 27 (2.5) | 28 (3.3) | 33 (3.5) | 26 (3.2) | 28 (2.9) | 31 (3.7) | 18 (1.9) | 20 (2.3) |
| Extreme living condition | Yes | 417 (33.4) | 438 (42.1) | 361 (34.4) | 345 (41.1) | 338 (36.0) | 363 (43.8) | 348 (35.8) | 367 (44.2) | 335 (35.6) | 369 (43.9) |
| Violence | Yes | 246 (19.7) | 172 (16.5) | 212 (20.2) | 128 (15.3) | 190 (20.2) | 129 (15.5) | 194 (19.9) | 128 (15.4) | 183 (19.5) | 120 (14.3) |
| Imprisonment/kidnapping | Yes | 225 (18.0) | 141 (13.6) | 200 (19.1) | 117 (13.9) | 173 (18.4) | 115 (13.9) | 172 (17.7) | 106 (12.8) | 170 (18.1) | 106 (12.6) |
| Serious injury | Yes | 253 (20.3) | 155 (14.9) | 223 (21.3) | 119 (14.2) | 204 (21.7) | 124 (15.0) | 202 (20.7) | 126 (15.2) | 195 (20.7) | 125 (14.9) |
| Murder/disappearance of family | Yes | 224 (18.0) | 197 (18.9) | 193 (18.4) | 157 (18.7) | 174 (18.5) | 159 (19.2) | 182 (18.7) | 162 (19.4) | 165 (17.5) | 165 (19.6) |
| Torture | Yes | 204 (16.36) | 133 (12.7) | 171 (16.3) | 103 (12.2) | 153 (16.3) | 100 (12.1) | 157 (16.1) | 103 (12.3) | 144 (15.3) | 97 (11.6) |
